# POLICY SERIES: GSA CONGRESSIONAL UPDATE

**DOI:** 10.1093/geroni/igad104.1095

**Published:** 2023-12-21

**Authors:** Patricia D’Antonio, Brian Lindberg

## Abstract

This popular annual session will provide cutting-edge information on what the 118th Congress has and has not accomplished to date, and what may be left for end of the First Session. Speakers will discuss key issues such as ending of public health emergency, Social Security, Medicare, Medicaid, and the Older Americans Act.

